# Supplementary material for: Assessment of COVID-19 Vaccine Acceptance and Its Associated Factors during the Crisis: A Community-Based Cross-Sectional Study in Benin
Source: Vaccines (Basel). 2023 Jun 16;11(6):1104. doi: 10.3390/vaccines11061104 (PMC10305180; doi:10.3390/vaccines11061104)
Supplement: Supplementary file 1 [file vaccines-11-01104-s001.zip › vaccines-2422785-supplementary.pdf]

Supplementary files S1 : Questionnaire

Session A. Identification du ménage

1. Date automatique de la visite /__/__/ /__/__/ /2/0/2/__/
2. Date manuel de la visite /__/__/ /__/__/ /2/0/2/__/
3. Nom de l'enquêteur ayant déroulé le questionnaire (liste déroulante)
4. Département : /________________________________/
5. Commune : /________________________________/
6. Arrondissement : /________________________________/
7. Village/Quartier : /________________________________/
8. Numéro du ménage : confère liste manuscrite
9. Identifiant du ménage (générer automatiquement par combinaison de la commune
10. Statut du ménage
    - a) Membres présents
    - b) Semble habité, mais personne n'est présent
    - c) Vacant (déménagement

Note : Si b) ou c), collecter vos coordonnées GPS et finaliser le questionnaire

11. Est-ce le chef de ménage ou le représentant du chef de ménage a donné son consentement écrit pour participe à l'enquête?
    - a) Oui, a donné son consentement
    - b) Non, a refusé
    - c) Non, n'est pas en mesure de répondre en ce moment
    - d) Non, chef de ménage absent et aucun adulte disponible en ce moment

Note : Si b) ou c) ou d) pour Q4, collecter vos coordonnées GPS et finaliser le questionnaire

11.1. Si non (option b), dire pourquoi le chef de ménage ou le représentant du chef de ménage a refusé de participer à l'enquête
    - a) Refuse, n'est pas intéressé
    - b) Expérience antérieure négative
    - c) Ne souhaite pas fournir des informations détaillées sur le ménage
    - d) Refuse en raison de préoccupations liées aux risques, et à la confidentialité
    - e) Ne souhaite pas signer le formulaire de consentement
    - f) Pas en mesure de donner son consentement
    - g) Autre, préciser

12. Qui est le répondant aux questions de l'enquête
    - a) Chef de ménage
    - b) Femme du chef de ménage
    - c) Autre membre du ménage
    - d) Autre, préciser

Session B. Informations démographiques du ménage

13. Sexe du chef de ménage ou de son répond (adulte) /__/ Féminin /__/Masculin

14. Age du répondant (chef de ménage) en années /__//__/

14.1. Si pas possible de donner l'âge exacte, donner un intervalle (en années)

a) 18-29 ans
b) 30-39 ans
c) 40-49 ans
d) 50-59 ans
e) 60 ans et plus

15. Quel est l'ethnie du chef de ménage (fonction des zones, à réajuster)

**Littoral**<br>a) Fon<br>b) Aizo<br>c) Goun<br>d) Yoruba<br>e) Adja<br>f) Autre, préciser	**Ouémé**<br>a) Goun<br>b) Yoruba<br>c) Fon<br>d) Nago<br>e) Mahi<br>f) Autre, préciser
**Atlantique**<br>a) Fon<br>b) Aizo<br>c) Toffin<br>d) Mahi<br>e) Adja<br>f) Autre, préciser	**Atacora**<br>a) Dendi<br>b) Batonou<br>c) Berba<br>d) Fon<br>e)<br>f) Autre, préciser

16. Type de zone

a) Urbain
b) Semi-rural
c) Rural

17. Statut marital

a) Célibataire
b) Mariée (tout type de mariage, même vivant maritalement)
c) Divorcée
d) Veuf
e) Refuse de répondre
f) Autre, préciser

18. Personnes vivants dans le ménage

a) Enfant (<18 ans) /__/ Oui /__/Non

b) Individus de plus de 65 ans /__/ Oui /__/Non

c) Individus avec des maladies chroniques /__/ Oui /__/Non

d) Non applicable /__/ Oui /__/Non

19. Éducation du chef de ménage ou du répondant

a) Pas d'éducation

b) Primaire incomplet

c) Primaire complet (CEP)

d) Secondaire incomplet

e) Secondaire complet (BEPC)

f) Secondaire supérieur incomplet

e) Secondaire supérieur complet (BAC)

f) Études supérieures

e) Ne sait pas

f) Ne veut pas répondre

g) Autre, préciser

20. Occupation du chef de ménage

a) Travaux de ménage

b) Agriculture

c) Pêche

d) Grand commerçant (Ex: vente en gros, pharmacie, SOBEBRA)

e) Petit commerçant (Ex: vente en détail, photocopie)

f) Travailleur dans les usines

g) Gardien de sécurité

h) Transport (Ex: Taxi-voiture, Taxi-moto)

i) Restaurant/Bar

j) Travailleur manuel (Ex: menuiser, soudeur, mécanicien, tailleur, coiffeur)

k) Agent administrative du public ou privée

l) Aucune

o) Autre, préciser

p) Ne sait pas

q) Refuse de répondre

21. Revenu mensuel net approximatif du ménage

a) < 50.000 FCFA

b) 51.000 à 150.000 FCFA

c) 150.001 à 250.000 FCFA

d) > 250.000 FCFA

e) Aucun
f) Occasionnel
g) refus de répondre

22. Principale religion dans le ménage
a) Christianisme
b) Islam
c) Vodoun
d) Aucune
e) Ne sait pas
f) Refuse de répondre
e) Autre, préciser

Session C. Caractéristiques de l’habitat

23. Observer le matériau principal du sol de la maison
a) Terre/sable/boue/argile
b) Fumier
c) Planches de bois
d) Palme/Bambou
e) Brique
f) Pierre
g) Parquet/Bois poli
h) Carreaux de céramique
i) Ciment
j) Pierre polie/Marbre/Granit
k) Autre, préciser

24. Observer le matériau principal du toît de la maison
a) Pas de toit
b) Toit en paille/feuille de cocotier/roseaux/herbe
c) Toit en plastique/Polythene
d) Tôle/Zinc
e) Bois
f) Ciment/dalle
g) Tuiles
h) Ardoise
i) Autre (préciser)

25. Observer le matériau principal des murs extérieurs de la maison
a) Terre/sable
b) Planches en bois

c) Palmes/Bambou
d) Briques/Pierre
e) Parquet ou bois ciré
f) Carrelage
g) Ciment
h) Autre (à préciser)

Session D. Bien disponibles dans le ménage

26. Possession de ce qui suit. Répondre Oui/Non
/__/ Vélo
/__/ Mobylette
/__/ Voiture
/__/ Charrette / Charrue
/__/ Électricité (SBEE)
/__/ Adduction d'eau (SONEB)
/__/ Groupe électrogène
/__/ Ventilateur
/__/ Télévision
/__/ Radio
/__/ Réfrigérateur / congélateur
/__/ Bœuf
/__/ Mouton / chèvre
/__/ Cuisinière
/__/ CD/DVD appareil
/__/ Ordinateur
/__/ un divan/Canapé
/__/ Matelas
/__/ Lampe à énergie solaire
/__/ Ventilateur de plafond
/__/ Lit
/__/ Panneau solaire
/__/ Téléphone portable
/__/ Ce ménage possède-t-il du bétail, des troupeaux, d'autres animaux de ferme ou de la volaille?

Session E. Sélection du participant du ménage

27. Combien de personnes vivent dans le ménage (résident, vivant/pas décédés) /__//__/

28. Combien de personnes du ménage présents ont plus de 12 ans ? /__//__/
    28.1. Donner le nom, prénoms de chacune de personnes présentes

28.2. Donner l'âge de chacune de personnes ?
28.3. Donner le sexe de chacune des personnes ?
28.4. Est-ce que chacune des personnes résident dans le ménage Oui/NOn
28.5. Est-ce que chacune des personnes est présente ou pas ? Oui/Non

Note : Sélection aléatoire d'une personne parmi ceux qui sont présents.

29. Est-ce que la personne sélectionnée a donné son consentement ? Oui/Non

NB : Si non à la question 29) prendre les coordonnées GPS et finaliser le questionnaire

Session F. Histoire médicale ou antécédents

30. Est-ce que la personne sélectionnée est différente du chef du ménage ou son représentant /__/ Oui /__/Non

Note : Si non, les questions 31 à 37 ne doivent pas apparaître

31. Sexe de la personne sélectionnée /__/ Féminin /__/Masculin

32. Age de la personne sélectionnée en années /__//__/
33. Si pas possible de donner l'âge exacte, donner un intervalle (en années)
    a) 18-29 ans
    b) 30-39 ans
    c) 40-49 ans
    d) 50-59 ans
    e) 60 ans et plus

34. Éducation de la personne sélectionnée
    a) Pas d'éducation
    b) Primaire incomplet
    c) Primaire complet (CEP)
    d) Secondaire incomplet
    e) Secondaire complet (BEPC)
    f) Secondaire supérieur incomplet
    e) Secondaire supérieur complet (BAC)
    f) Études supérieures
    e) Ne sait pas
    f) Ne veut pas répondre
    g) Autre, préciser

35.Occupation de la personne sélectionnée

a) Travaux de ménage
b) Agriculture
c) Pêche
d) Grand commerçant (Ex: vente en gros, pharmacie, SOBEBRA)
e) Petit commerçant (Ex: vente en détail, photocopie)
f) Travailleur dans les usines
g) Gardien de sécurité
h) Transport (Ex: Taxi-voiture, Taxi-moto)
i) Restaurant/Bar
j) Travailleur manuel (Ex: menuiser, soudeur, mécanicien, tailleur, coiffeur)
k) Agent administrative du public ou privée
l) Aucune
o) Autre, préciser
p) Ne sait pas
q) Refuse de répondre

36. Revenu mensuel net approximatif de la personne sélectionnée

a) < 50.000 FCFA
b) 51.000 à 150.000 FCFA
c) 150.001 à 250.000 FCFA
d) > 250.000 FCFA
e) Aucun
f) Occasionnel
g) refus de repondre

37. Religion de la personne sélectionnée

a) Christianisme
b) Islam
c) Vodoun
d) Aucune
e) Ne sait pas
f) Refuse de répondre
e) Autre, préciser

38. Avez-vous des antécédents de la personne sélectionnée

a) Diabètes	/__/ Oui /__/Non
b) Hypertension artérielle	/__/ Oui /__/Non
c) Antécédents cardiaques	/__/ Oui /__/Non
d) Maladie thromboembolique	/__/ Oui /__/Non
e) Tumeurs malignes/cancers	/__/ Oui /__/Non

f) Affections respiratoires (asthme, Bronchopathie obstructive) /__/ Oui /__/Non
g) Immunodéficience ou maladie auto-immune /__/ Oui /__/Non
h) Insuffisance rénale /__/ Oui /__/Non
i) Autre, préciser /__/ Oui /__/Non

39. Êtes-vous un fumeur actif ?
a) Non
b) Oui, cigarettes
c) Oui, pipe artisanale
d) Oui, cigares
e) Oui, cigarette électronique

40. Avez-vous des allergies médicamenteuses /__/ Oui /__/Non
41. Avez-vous d'autres allergies (alimentaires ?) /__/ Oui /__/Non

Session G. Connaissance et attitudes

42. Connaissances, mode de transmission
Le nouveau coronavirus peut être transmis directement par (*Veuillez en sélectionner autant que possible)*
a) Toux
[ ] Vrai [ ] Faux [ ] Ne sait pas
b) Contact avec des surfaces infectées
[ ] Vrai [ ] Faux [ ] Ne sait pas
c) Consommation de produits laitiers et de viande contaminés
[ ] Vrai [ ] Faux [ ] Ne sait pas
d) Contact avec des personnes infectées (poignée de main, étreinte, baiser)
[ ] Vrai [ ] Faux [ ] Ne sait pas

43. Connaissances des symptômes
Lesquels des éléments suivants peuvent être des symptômes du nouveau coronavirus ?
(*Veuillez en sélectionner autant que possible)*
a) Fièvre
[ ] Vrai [ ] Faux [ ] Ne sait pas
b) Toux
[ ] Vrai [ ] Faux [ ] Ne sait pas
c) Essoufflement
[ ] Vrai [ ] Faux [ ] Ne sait pas
d) Mal de gorge
[ ] Vrai [ ] Faux [ ] Ne sait pas
e) Nez qui coule ou qui est bouché
[ ] Vrai [ ] Faux [ ] Ne sait pas
f) Douleurs musculaires ou corporelles
[ ] Vrai [ ] Faux [ ] Ne sait pas
g) Maux de tête

[ ] Vrai [ ] Faux [ ] Ne sait pas

h) Fatigue
   [ ] Vrai [ ] Faux [ ] Ne sait pas
i) Diarrhée
   [ ] Vrai [ ] Faux [ ] Ne sait pas
j) Perte du goût et de l'odorat
   [ ] Vrai [ ] Faux [ ] Ne sait pas

44. Prévention - comportements propres

Parmi les mesures suivantes, quelles sont celles que vous avez prises pour prévenir l'infection par le nouveau coronavirus ? (*Veuillez indiquer pour toutes les mesures ci-dessous si vous les avez déjà prises)*

a) Se laver les mains pendant au moins 20 secondes
   [ ] Oui [ ] Non [ ] Non applicable
b) Éviter de se toucher les yeux, le nez et la bouche avec des mains non lavées
   [ ] Oui [ ] Non [ ] Non applicable
c) Utilisation de désinfectants pour se nettoyer les mains lorsque l'eau et le savon ne sont pas disponibles pour le lavage des mains
   [ ] Oui [ ] Non [ ] Non applicable
d) Rester à la maison quand on est malade ou quand on a un rhume
   [ ] Oui [ ] Non [ ] Non applicable
e) Utilisation de compléments alimentaires à base de plantes
   [ ] Oui [ ] Non [ ] Non applicable
f) Se couvrir la bouche et le nez quand on tousse ou éternue
   [ ] Oui [ ] Non [ ] Non applicable
g) Le port d'un masque facial
   [ ] Oui [ ] Non [ ] Non applicable
h) Distanciation physique (maintenir une distance minimale de 2 mètres entre vous et les autres personnes extérieures à votre foyer)
   [ ] Oui [ ] Non [ ] Non applicable

45. Comportements

Avez-vous fait ce qui suit pendant la pandémie... ?

a) Éviter les grands rassemblements publics, par exemple les événements religieux ou sociaux
   [ ] Oui [ ] Non [ ] Ne sait pas
b) Éviter d'aller chez le médecin pour des questions qui pourraient être reportées, par exemple une vaccination ou un examen
   [ ] Oui [ ] Non [ ] Ne sait pas
c) Éviter d'aller chez le médecin lorsque mon enfant et/ou moi-même avions de la fièvre
   [ ] Oui [ ] Non [ ] Ne sait pas
d) Éviter de rendre visite à la famille ou aux amis
   [ ] Oui [ ] Non [ ] Ne sait pas
e) Acheter des médicaments dont j'ai entendu parler et qui sont bons pour le traitement du COVID-19

[ ] Oui [ ] Non [ ] Ne sait pas

f) Acheter des équipements de protection individuelle (masques, gants)

[ ] Oui [ ] Non [ ] Ne sait pas

Session H. Expérience avec l'infection à la COVID-19

46. Avez-vous été infecté par la COVID-19 ?

a) Oui
b) Non
c) Ne sait pas
d) Refuse de répondre

46.1. Si oui Q.46, pouvez-vous nous dire la sévérité des symptômes

a) Légère (ne modifie pas les habitudes quotidiennes)
b) Modérée (modifie les habitudes quotidiennes)
c) Sévère (hospitalisation)
d) Ne sait pas

46.2. Si oui Q46. Souffrez-vous encore de signes résiduels ?

a) Oui
b) Non
c) Ne sait pas

47. A quel point avez-vous peur d'être infecté/malade ou réinfecté par la COVID-19 sur une échelle de 0 à 10 (zéro étant non aucune peur et 10 avoir très peur) /__//__/

48. Connaissez-vous un ami ou un membre de votre famille qui a été hospitalisé ou décédé d'une infection à la COVID-19 ? /__/ Oui /__/Non

49. Où avez-vous entendu les information sur les vaccins COVID-19 ?

a) Radio
b) Télévision
c) Journal papier
d) Réseaux sociaux (Whatsaap, facebook, etc.)
e) Internet
f) Centre de santé
g) Sensibilisation des autorités du village/quartier
h) Espaces religieux (églises, etc)
i) Bouches à oreilles
j) Autre, préciser

Session I. Pratiques et croyances

50. Quelle est votre pratique actuelle avec les médicaments en général ?

a) Habituellement, je les prends en cas de besoin (automédication, etc)
b) Uniquement sur prescription médicale
c) Je n'aime pas du tout prendre des médicaments
d) Autre, préciser

51. Êtes-vous totalement opposé à la vaccination en tant que concept ?
a) Oui
b) Non
c) Ne sait pas
d) Refuse de répondre

52. Êtes-vous vacciner contre la COVID-19 ?
a) Oui
b) Non
c) Non pas encore
d) Refuse de répondre

52.1. Si « non pas encore » à la question Q52
Comptez- vous, vous faire vacciner d'ici
a) Les jours à venir
b) Les semaines à venir
c)Les mois à venir
d) un jour dans les années à venir
e) si seulement je suis obligé
f) Jamais de la vie
g) Je ne sais pas
h) Refuse de répondre

52.2. Si oui à la Q52, quel type de vaccin avez-vous reçu ?

a) Astra-zénéca (Covishild)	/__/ Oui /__/Non
b) Sinovac (chinois)	/__/ Oui /__/Non
c) Johnson (Jansen)	/__/ Oui /__/Non
d) Fizer	/__/ Oui /__/Non
e) Moderna	/__/ Oui /__/Non
f) Spoutnik (Ruissie)	/__/ Oui /__/Non
g) Ne sait pas	/__/ Oui /__/Non
h) Autre, préciser	/__/ Oui /__/Non

52.3. Si oui à la Q52, combien de doses avez-vous reçu ? /__/

52.3.1. Si dose >=1, préciser les dates

Date dose 1 : /__/__/ /__/__/ /2/0/2/__/
Date dose 2 : /__/__/ /__/__/ /2/0/2/__/
Date dose 3 : /__/__/ /__/__/ /2/0/2/__/

52.4. L'information a-t-elle été confirmé par le carnet de vaccination ? (demander à voir le carnet)

a) Oui
b) Non, carnet non vu
c) Non, refuse de montrer son carnet
d) Non, carnet non accessible actuellement

52.5. Si « oui » ou « non pas encore » à la Q52, s'il vous plaît dites-nous pourquoi vous vous êtes déjà vacciner ou pourquoi vous voulez vous vacciner

a) Parce que cela est obligatoire pour voyager à l'étranger
b) Parce que cela est obligatoire pour aller au travail
c) Parce que je veux que la pandémie se termine rapidement
d) Parce que je veux me protéger contre l'infection à la COVID-19
e) Parce que je veux protéger ma famille et mes proches contre l'infection à la COVID
f) Parce que j'ai peur de la gravité et des complications de la COVID-19
g) Parce que je veux reprendre mes activités professionnelles
h) Parce que je fais partie d'un groupe à haut risque
i) Parce qu'il est recommandé par les médecins
j) Parce qu'il est recommandé par le ministère de la santé
k) Parce qu'il est recommandé par l'Organisation Mondiale de la Santé
l) Autre, préciser

52.6. Si non à la Q52, accepterez-vous vous faire vacciner ?

a) Oui
b) Non
c) Ne sait pas
d) Refuse de répondre

52.6.1. Si non ou ne sait pas à la Q52.6, Puisque vous ne souhaitez pas ou hésitez encore à vous faire vacciner, veuillez préciser vos raisons ?

a) Je pense que le vaccin n'est peut-être pas efficace
b) Je pense que le vaccin pourrait ne pas être efficace avec les nouvelles souches
c) Je suis préoccupé par les effets secondaires à court terme que les vaccins COVID-19 peuvent causer
d) Je suis préoccupé par les effets des nouvelles technologies (ARNm) utilisées
e) Je n'ai pas besoin d'être vacciné car je suis jeune
f) Je n'ai pas besoin d'être vacciné car j'ai déjà été infecté par le COVID-19 et cela me

confère assez d'immunité

g) Je ne veux pas me faire vacciner car je pense que le temps alloué aux essais cliniques des vaccins est trop court

h) Je ne veux pas me faire vacciner car je pense que la vaccination contre le COVID-19 est un complot (5G, implantation de « micro-puces »)

i) Je ne veux pas me faire vacciner parce que je suis préoccupé par la capacités du gouvernement à maintenir la qualité du vaccin

j) Je ne veux pas me faire vacciner pour des raisons religieuses

k) Autre, préciser

53. Si vous avez décidé de ne pas vous faire vacciner ou si vous hésitez encore, que devez-vous savoir pour accepter le vaccin COVID-19? (plusieurs réponses peuvent s'appliquer)

a) J'aimerais avoir plus d'information à ce sujet

b) J'attendrais de voir les résultats objectifs

c) Si j'ai le droit de choisir la marque du vaccin

d) Si un leader ou un chef religieux se fait vacciner

e) Si une autorité du gouvernement se fait vacciner

f) Non applicable

g) Autre, préciser

54. Pensez-vous que la vaccination a pu affecter votre croyance sur les vaccins autre que celui de la COVID-19? /__/ Oui /__/Non

55. Maintenant, laisseriez-vous ou conseillerez-vous votre famille se faire vacciner pour les autres vaccins PEV et hors-PEV autre que celui de la COVID-19 ? /__/ Oui /__/Non

Session J. Autres commentaires

Si vous avez d'autres commentaires, ajouter les ici.
